# Intravenous metastasis of unexpected uterine sarcoma in the context of uterine fibroids: case report and literature review

**DOI:** 10.3389/fonc.2024.1354032

**Published:** 2024-02-15

**Authors:** Wenying Huang, Tianwei Zhang, Hui Wang, Zhengchun Liu, Peiling Zhai, Xinbo Wang, Shuai Wang

**Affiliations:** ^1^ School of Clinical Medicine, Weifang Medical University, Weifang, Shandong, China; ^2^ Department of Hematology and Radiotherapy, Zibo 148 Hospital, Zibo, Shandong, China; ^3^ Department of Gynecology, Affiliated Hospital of Weifang Medical University, Weifang, Shandong, China; ^4^ Department of Radiotherapy, School of Medical Imaging, Affiliated Hospital of Weifang Medical University, Weifang Medical University, Weifang, Shandong, China

**Keywords:** high-grade endometrial stromal sarcoma (HG-ESS), *MED12*, venous metastasis, unexpected uterine sarcoma, low-grade endometrial stromal sarcoma (LGESS)

## Abstract

**Objective:**

Endometrial stromal tumors are rare and complex mesenchymal tumors that often present with clinical symptoms similar to uterine leiomyomas. Due to their atypical nature, they are prone to be misdiagnosed or overlooked by healthcare professionals. This study presents a case report of an incidentally discovered endometrial stromal sarcoma with venous metastasis, which was initially misdiagnosed as a uterine leiomyoma. In addition, this study reviews previously documented cases of similar tumors.

**Case report:**

During a routine medical examination in 2016, a 50-year-old woman was diagnosed with uterine fibroids. In June 2020, she began experiencing moderate, irregular vaginal bleeding. Nevertheless, a histopathological examination indicated an endometrial stromal sarcoma with a striking amalgamation of both low-grade and high-grade features. Molecular analysis identified a rare *MED12* gene mutation. The patient underwent total hysterectomy, bilateral salpingectomy, and resection of the metastatic lesions. Postoperative management included radiotherapy, chemotherapy, and hormone therapy. After completion of chemotherapy, the patient was followed up for 27 months with no evidence of tumor recurrence.

**Conclusion:**

This case report highlights the importance of pathological, immunohistochemical, and molecular aspects of this rare tumor involving the inferior vena cava and showing the presence of atypical gene mutations. The successful treatment outcome further emphasizes the importance of advances in diagnostic modalities for managing rare tumors like this.

## 1 Introduction

Endometrial stromal and associated neoplasms are a rare and complex group of mesenchymal tumors that develop within the uterus. Endometrial stromal sarcoma (ESS) is an aggressive tumor arising from endometrial stromal cells, representing about 1% of uterine malignancies and <10% of uterine stromal tumors (1). Most patients with uterine sarcoma have no specific symptoms, with approximately 25% of the patients being asymptomatic or presenting with symptoms similar to uterine fibroids (2). In some cases, uterine sarcoma is found during intraoperative frozen section examination or postoperative pathological diagnosis after myomectomy or total hysterectomy, which is known as “unexpected uterine sarcoma” in some academic circles (3). In the most recent classification by the World Health Organization (WHO, 2020), endometrial stromal and associated tumors have been classified into four distinct groups: endometrial stromal nodule (NST), low-grade endometrial stromal sarcoma (LGESS), high-grade endometrial stromal sarcoma (HGESS), and undifferentiated uterine sarcoma (UUS) (4). This classification is based on gross morphological characteristics, histological observations, immunophenotype, and the expression of genetic abnormalities associated with the tumor.

The patient was diagnosed with uterine fibroids during a routine medical examination in December 2016. Because of the absence of any discomfort or irregular vaginal bleeding, the patient did not maintain regular follow-ups or seek further medical consultation. In June 2020, the patient attributed the irregular vaginal bleeding to menopausal manifestations and opted to forego medical attention. In December 2020, the patient finally sought care at our institution, where she underwent a diagnostic hysteroscopy. The postoperative diagnosis was LGESS which was an incidental finding of uterine sarcoma. The National Comprehensive Cancer Network (NCCN) guidelines (5) recommend advancing imaging modalities to gain a more thorough understanding of the lesion and its anatomical relationship with surrounding pelvic and abdominal organs. This helps to assess the lymph node involvement and the presence of suspicious extrauterine metastases. In this case, unsuccessful removal of the intrauterine device because of the adhesions contraindicates the use of magnetic resonance imaging (MRI) for the investigation. Therefore, we adopted a combination of plain and contrast-enhanced computed tomography (CT) scans as an alternative. An abdominal CT scan of the woman showed adenomyosis involving both adnexa, multiple leiomyomas with partial cystic changes, and significant dilatation of the bilateral uterine arteries and veins. Furthermore, dilation was noticeable in the inferior vena cava, left iliac vein, and pelvic veins, indicating the possibility of venous leiomyoma. Two weeks following the hysteroscopic biopsy, she underwent major abdominal surgery including total hysterectomy, bilateral salpingectomy, and resection of tumor from the inferior vena cava, the left common iliac vein, and the left internal iliac vein. The conclusive pathological diagnosis revealed the presence of an HGESS with intravascular metastases.

The patient was diagnosed with uterine fibroids during a routine medical examination in December 2016. Despite the absence of any discomfort or irregular vaginal bleeding, the patient did not maintain regular follow-ups or seek further medical consultation. It was not until June 2020 when irregular vaginal bleeding occurred that the patient attributed this symptom to menopausal manifestations and opted to forego medical attention. Only in December 2020 did the patient finally seek care at our institution, where she underwent a diagnostic hysteroscopy. The postoperative diagnosis was low-grade endometrial stromal sarcoma (LGESS), which was an incidental finding of uterine sarcoma. The National Comprehensive Cancer Network (NCCN) guidelines (5) recommend enhancing imaging examinations to gain a more thorough understanding of the lesion and its anatomical relationship with surrounding pelvic and abdominal organs. This helps to assess lymph node involvement and the presence of suspicious extrauterine metastases. In this case, due to the unsuccessful removal of the intrauterine device caused by adhesions, magnetic resonance imaging (MRI) is contraindicated. Therefore, we have chosen a combination of plain and contrast-enhanced computed tomography (CT) scans as an alternative. Abdominal CT scan of the woman showed adenomyosis involving both adnexa, multiple leiomyomas with partial cystic changes and significant dilatation of bilateral uterine arteries and veins. Furthermore, dilation was noticeable in the inferior vena cava, left iliac vein and pelvic veins, indicating the possibility of venous leiomyoma. Two weeks following the hysteroscopic biopsy, she underwent major abdominal surgery including total hysterectomy, bilateral salpingectomy, resection of tumor from the inferior vena cava, resection of tumor from the left common iliac vein and resection of tumor from the left internal iliac vein. The conclusive pathological diagnosis revealed the presence of a high-grade endometrial stromal sarcoma (HGESS) with intravascular metastases.

## 2 Methods and results

### 2.1 Case presentation

**Figure 1:**
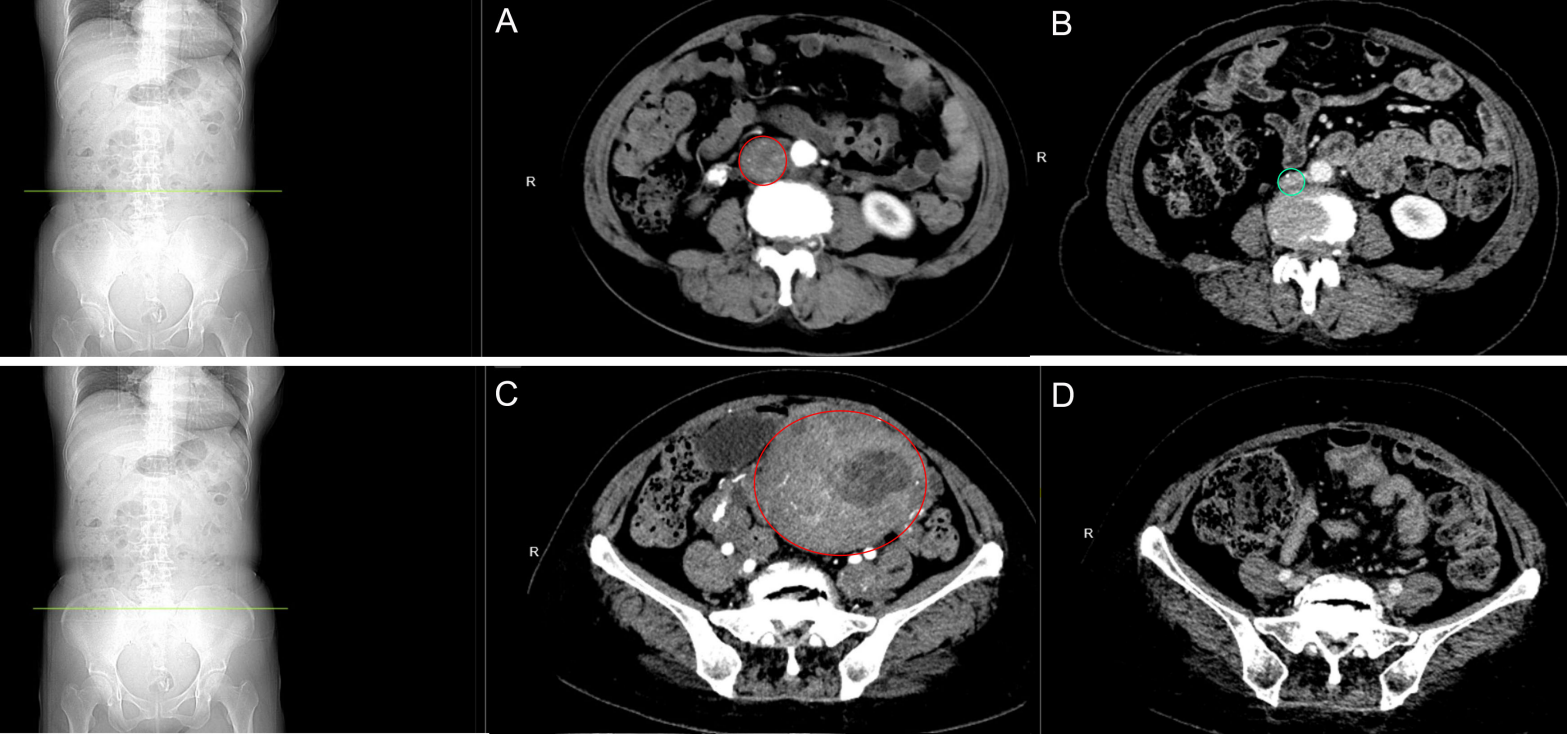
Abdominal CT scan. **(A)** Preoperative image of the inferior vena cava: The inferior vena cava was observed to be dilated, suggestive of smooth muscle leiomyoma. **(B)** Follow-up image of the inferior vena cava: Following the completion of chemotherapy after 27 months, no abnormalities were detected. **(C)** Preoperative image of the uterus: Multiple smooth muscle leiomyomas are present in the uterus and bilateral adnexa, with partial cystic degeneration. **(D)** Follow-up pelvic examination: 27 months after chemotherapy, changes indicative of postoperative alterations in the uterus were observed.

The patient, a 50-year-old female, was diagnosed with uterine fibroids during a routine medical examination in 2016. In June 2020, she experienced moderate-intensity irregular vaginal bleeding, occasionally accompanied by dark red blood clots. In December 2020, the patient came to our hospital for consultation and underwent a gynecological ultrasound examination. The ultrasound revealed an anteverted uterus measuring approximately 10.5×8.3×9.0 cm. Multiple hypoechoic lesions were found within the myometrium. The largest fibroid, approximately 2.6×1.4 cm in size, was found in the anterior wall. Additionally, a 7.3×5.5×7.0 cm solid cystic echo was observed in the anterior wall muscle. These findings suggest the presence of multiple uterine fibroids and possible changes in the endometrium. Moreover, it was noted that the intrauterine contraceptive device had descended. During the gynecological examination, a significant amount of dark red blood clot tissue was found in the vagina of the patient, accompanied by a foul-smelling discharge. The cervix was difficult to expose, and the uterus appeared to be the size of a 4-month-old pregnancy. It had a firm consistency, irregular shape, and was mobile. No tenderness was observed upon palpation, and no abnormalities were detected in the bilateral adnexa. The patient denied any family history of similar diseases. The findings of auxiliary examinations revealed the levels of tumor markers as follows: carbohydrate antigen 125 (CA125) 22.48 U/mL (normal range: 0-35 U/mL), carbohydrate antigen 19-9 (CA19-9) <0.600 U/mL (normal range: 0-27 U/mL), and human epididymis protein 4 (HE4) 73.69 pmol/L (normal range: 0-140 pmol/L). Owing to the enlarged uterus and poor exposure of the cervix, diagnostic dilation and curettage was difficult to perform, severely reducing the accuracy of the biopsy. Therefore, on December 21, 2020, an intrauterine mass biopsy was performed on the patient after a comprehensive evaluation. The postoperative pathological examination revealed a “spindle cell tumor of the uterine cavity,” with immunohistochemistry indicating a tendency toward an LGESS. To further assess the interplay between the lesion and adjacent tissues, the patient underwent chest and abdominal CT scans. The abdominal CT revealed adenomyosis along with multiple leiomyomas, some of which exhibited partial cystic degeneration. There was substantial thickening of the bilateral vasculature, including the arterial and venous systems. In addition, there were lesions within the uterine cavity (**Figure 1A**), and there was evident dilation of the inferior vena cava, left iliac vein, and pelvic veins, indicative of venous leiomyoma (**Figure 1C**). The chest CT did not reveal any major abnormalities. The preoperative diagnosis revealed “LGESS” and “Intravascular tumor”. After a comprehensive evaluation of the patient’s condition, on January 4, 2021, she underwent a “complete abdominal hysterectomy with bilateral salpingo-oophorectomy and tumor resection from the inferior vena cava, left common iliac vein, and left internal iliac vein.” The diagnosis of “HGESS with intravascular metastases” was established based on the postoperative pathology findings.

### 2.2 Gross examination

**Figure 2:**
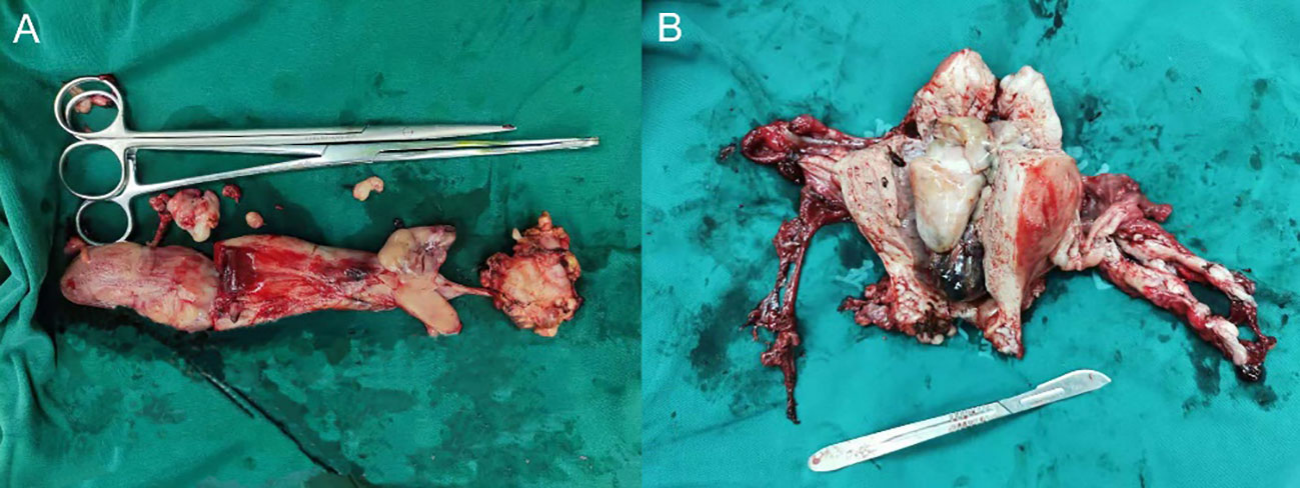
Gross examination. **(A)** Intravascular tumor tissue: a mass of grayish-white tissue measuring 5 × 3.5 × 1 cm in volume. **(B)** Uterus and bilateral adnexal tissue: The uterus has a volume of 14.5 × 10 × 7 cm, with a cervical length of 8.5 cm. The external cervical orifice is 5 cm in diameter and the internal cervical orifice is 2.5 cm in diameter. The anterior uterine wall is approximately 3 cm thick. A protruding mass with a total volume of 8 × 4 × 4 cm is present within the uterine cavity. The dimensions of the left ovary are 4 × 3 × 1.5 cm, and the fallopian tube has a length of 5 cm and a maximum diameter of 1 cm. The right ovary measures 3.5 × 2.5 × 1 cm, and the fallopian tube has a length of 6.5 cm and a maximum diameter of 0.8 cm.

In surgery, tumor emboli were palpable within the left common iliac vein and inferior vena cava, with margins extending approximately 2 cm above the renal veins. Grayish-white tumor tissue measuring 5 x 3.5 x 1 cm was successfully extracted from within the blood vessels (**Figure 2A**). Upon dissection of the enlarged uterus and adnexa (**Figure 2B**), the following findings were observed: the uterine volume measured 14.5 x 10 x 7 cm, the cervical length was 8.5 cm, the external cervical uterine diameter was 5 cm, and the internal cervical uterine diameter was 2.5 cm. The uterine wall anteriorly showed hypertrophy, measuring approximately 3 cm in thickness. The uterine cavity contained an elevated mass, which appeared grayish-white in cross-section and measured 8 x 4 x 4 cm. Additionally, multiple grayish-white nodules, ranging in diameter from 0.5 to 1.5 cm, were observed within the uterine wall. No significant abnormalities were identified in the bilateral adnexa.

### 2.3 Microscopic examination of tumor cells

**Figure 3:**
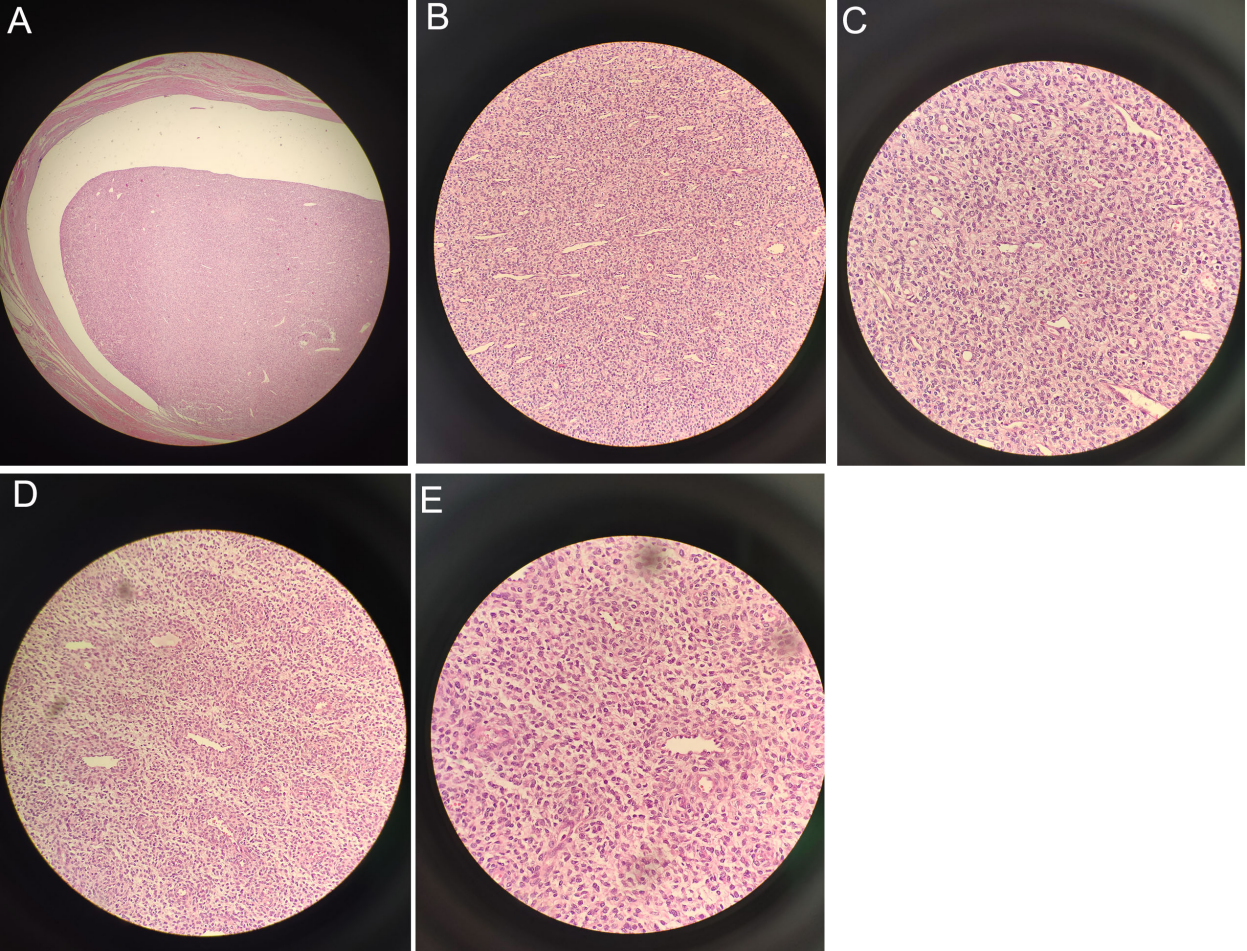
Under the microscope: **(A)** Localization of the intracavitary mass revealed distinct borders (X40). **(B)** Most of the cells appeared ovoid (X200). **(C)** Visible nuclear division with moderate-to-severe anisokaryosis (X400). **(D)** Tumor wrapped around small arteries in a whirlpool-like pattern (X200). **(E)** Consistent with high-grade endometrial stromal sarcoma (X400).

An Olympus (CX43) optical microscope was used for observation. Localization of the intracavitary mass showed distinct borders (**Figure 3A** X40). In certain areas, the cells were densely packed and showed active growth. The tumor tissue invaded the deep muscular layer of the uterine wall, reaching the serosal surface, and extended into the cervical canal. In addition, tumor tissue was present at the cut end of the cervix with evidence of neural invasion and intravascular tumor thrombi. Cancer cells are abundant but irregular in size and shape, with most of them appearing ovoid (**Figure 3B** X200). The cytoplasm is either sparse or vacuolated and the nuclei are enlarged with an increased nuclear to cytoplasmic ratio. Moderate-to-severe anisokaryosis was observed with visible nuclear division (8/10 high-power fields) (**Figure 3C** X400). Within the mass, small arteries, thin-walled vessels, and numerous thick-walled vessels were observed. In addition, within the vasculature, the tumor was wrapped around the small arteries in a vortex-like pattern (**Figure 3D** X200). The results indicate conformity with HGESS (**Figure 3E** X400).

### 2.4 Immunohistochemistry

On December 21, 2020, the results of the biopsy of tumor tissue demonstrated partial positivity for CD10 and a Ki-67 proliferation index of 20-30%. They further exhibit positivity for Wilms Tumor 1 (WT-1) and smooth muscle actin (SMA), but negativity for h-Caldesmon staining.

**Figure 4:**
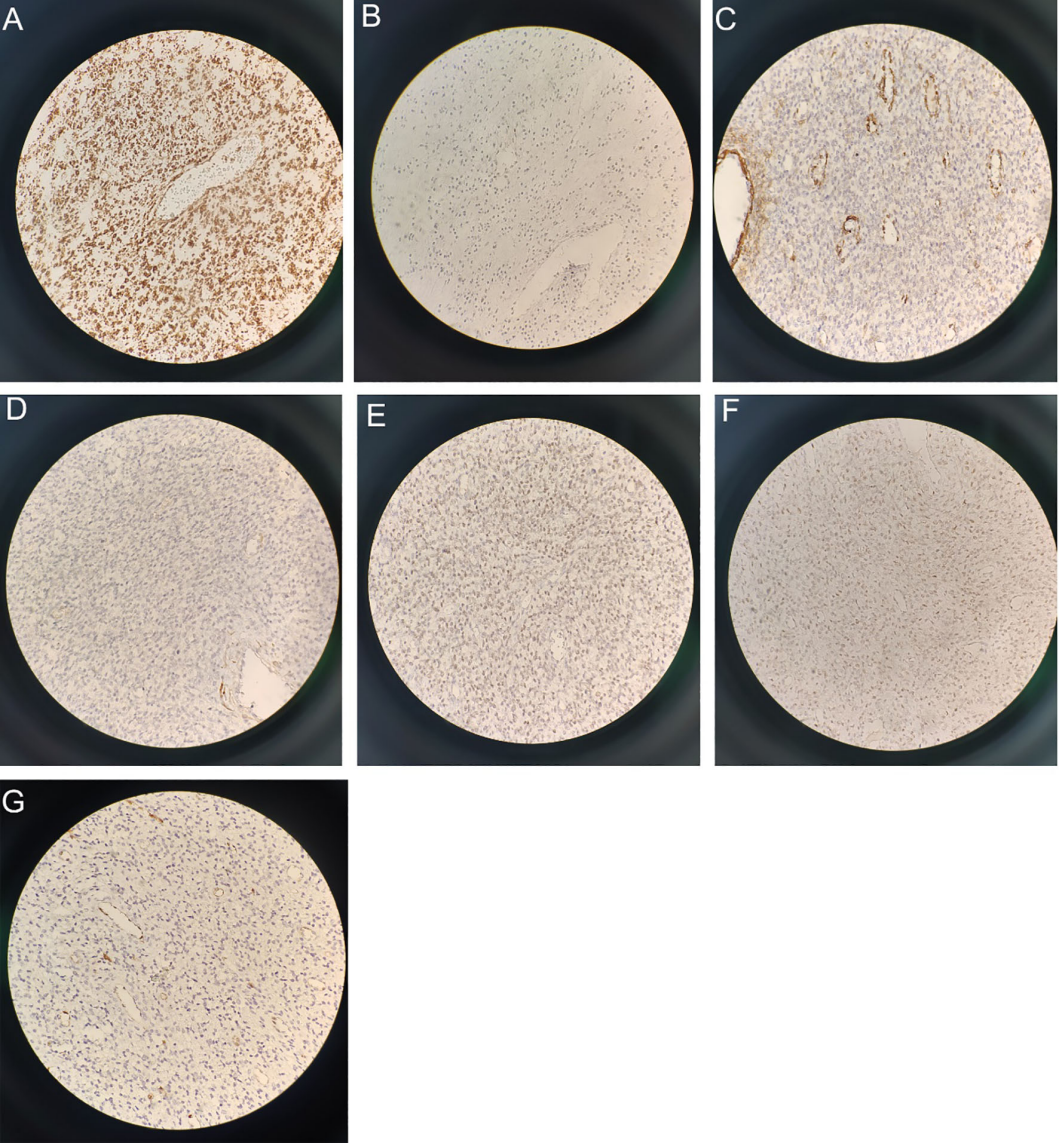
Immunohistochemical staining results (X400): **(A)** Immunohistochemical staining revealed CD10 positivity. **(B)** Ki-67 demonstrated a positive rate of approximately 40%. **(C)** SMA shows positivity. **(D)** h-Caldesmon staining is negative. **(E)** ER staining is positive. **(F)** PR staining exhibits weak positivity. **(G)** Cyclin D1 shows weak positivity.

On January 4, 2021, the tumor cells located within the uterine cavity displayed positive CD10 immunohistochemical staining results (**Figure 4A** X400), with an approximate 40% positive rate of Ki-67 (**Figure 4B** X400). In addition, the cells demonstrated positive SMA (**Figure 4C** X400), negative h-Caldesmon (**Figure 4D** X400), positive estrogen receptor (ER) (**Figure 4E** X400), and weakly positive progesterone receptor (PR) (**Figure 4F** X400) and cyclin D1 (**Figure 4G** X400). The WT-1 and Vimentin markers were positive whereas EMA and HMB45 were negative.

### 2.5 Next-generation sequencing testing

Next-generation sequencing (NGS) is used to extract and sequence tumor samples that are embedded in paraffin wax. The raw sequencing data is filtered and then subjected to bioinformatic analysis. The reference sequence used for NGS testing was GRCh37/hg19 (**Supplementary Figure 1**: Gene mutations). The detected types of gene variants included fusion mutations, point mutations, insertion/deletion mutations, and copy number variations. A frameshift mutation, p. T920Lfs*36, was discovered in the 20th exon of the *MED12* gene. The mutation identified was c.2757del (p. T920Lfs*36) and had a variant abundance of 40.38%. Furthermore, the *RB1* gene experienced a loss in copy number (CN) with a CN of 0.39. Finally, a missense mutation, p.Y251C (c.752A>G), was observed in the 3rd exon of the *VEGFA* gene with an abundance of 1.26%. Moreover, a heterozygous polymorphism, namely c.313A>G (p.I105V), was present in the *GSTP1* gene. However, the fusion genes associated with HGESS, including YWHAE-NUTM2A, YWHAE-NUTM2B, and ZC3H7B-BCOR were not detected in this case.

### 2.6 Treatment and follow-up results

According to the 2023 International Federation of Gynecology and Obstetrics (FIGO) staging criteria and treatment recommendations for uterine sarcoma (6, 7), the patient in this case was classified as stage IVB. The treatment involved a total abdominal hysterectomy with bilateral salpingo-oophorectomy, as well as the removal of a tumor from the inferior vena cava, the left common iliac vein, and the left internal iliac vein. Following the surgery, the patient is taking letrozole orally 2.5 mg daily. Moreover, one month after the surgery, the patient underwent intensity-modulated radiotherapy (IMRT) for a total of 25 times with a dose of 2 Gy each time, followed by post-loading radiotherapy for a total of 6 times with a dose of 5 Gy each time. The patient underwent six cycles of chemotherapy with ifosfamide (2 g on days 1-3 every 21 days), etoposide (60 mg on days 1-2 every 21 days), and cisplatin (30 mg on days 1-3 every 21 days) simultaneously. After completing systemic treatment, the patient will undergo routine physical examinations and repeat serum CA125 testing every 3 months for 3 years. The chest, abdomen, and pelvic CT scans will be conducted every 6 months. In case of suspicion of tumor metastasis, a comprehensive positron emission tomography (PET)-CT examination will be performed for further evaluation. As per the most recent follow-up, which occurred 27 months after the completion of chemotherapy, the echocardiography assessment indicated the absence of any focal mass within the ventricle. Furthermore, a follow-up CT scan of the abdomen and pelvis, enhanced with contrast, revealed no abnormal masses (**Figures 1B, D**).

## 3 Discussion

Endometrial stromal sarcoma is a rare tumor type, comprising only 0.2% of all malignant uterine tumors. Nevertheless, ESS represents approximately 7-25% of all uterine sarcomas (8). The annual incidence rate is 0.19 cases per 100,000 females, showing a gradual increase over the past decade. ESS is regarded as the second most prevalent uterine mesenchymal tumor after uterine leiomyoma (9),. The worldwide median age for the diagnosis of ESS diagnosis is 55 years (10). Most of the patients do not experience specific symptoms, and up to 25% of the patients may remain asymptomatic (2). Owing to the absence of clear clinical manifestations and the resemblance of symptoms to uterine fibroids, most patients with ESS are initially misdiagnosed to have uterine fibroids, leading to the term “unexpected uterine sarcoma” being applied in these cases. The diagnostic challenges associated with ESS are related to its morphological features, clinical behavior, and genetic abnormalities. The condition is categorized into two types based on these characteristics: LGESS and HGESS. According to a large-scale study, 86% of all ESS cases are LGESS, whereas the remaining 14% are categorized as HGESS (11). Nevertheless, the complexity and heterogeneity of these tumors go beyond this diagnostic classification.

LGESS is the dominant subtype of ESS. It is characterized by late recurrence and has an indolent nature. The tumor grows slowly and has a favorable prognosis. LGESS is typically identified within the uterus during an investigation of hysterectomy specimens (9). In this case report, the clinical presentation together with abdominal ultrasound and CT scans suggested the presence of uterine fibroids. However, postoperative pathology revealed the coexistence of both LGESS and HGESS. LGESS commonly presents in perimenopausal women and is rarely reported in young women and adolescents (12). Risk factors shared among these age groups include obesity, diabetes, early menarche, and the use of tamoxifen and estrogen medications (9, 13, 14). The woman in this case study had a BMI >28 kg/m^2^, indicating an obesity level, and a 7-year history of diabetes.

Pathologically, LGESS comprises uniform tumor cells with features resembling proliferative endometrial stroma. Its histological features include uniform and densely packed stromal cells accompanied by mild nuclear atypia (<5 per 10 high-power fields) and occasional mitotic activity. Immunohistochemically, positive staining can be observed for CD10, WT-1, vimentin, interferon-induced transmembrane protein 1 (IFITM1), ER, androgen receptor (AR), and PR. Nevertheless, Cyclin D1 and desmin yield negative or focally positive results. Some researchers have pointed out that the immunohistochemical profile of LGESS is characterized by positive staining for CD10, ER, and PR (15). LGESS can be distinguished based on the presence of IFITM1, providing a level of specificity in its detection (16). CD10 as a marker of mesenchymal tumors exhibits high sensitivity and is the most commonly used immunohistochemical antibody for distinguishing LGESS from HGESS (1). However, it is worth noting that in clinical practice, a few cases of LGESS may be negative, weakly positive, or focally positive for CD10. Therefore, positive CD10 staining alone does not suffice as a specific marker for LGESS. The diagnosis of LGESS requires a combination of CD10, cyclin D1, ER, PR, h-Caldesmon, SMA, and desmin. In certain cases, molecular genetic alterations can also establish a definitive diagnosis. Common fusion genes identified in LGESS comprise JAZF1-SUZ12, JAZF1-PHF1, EPC1-PHF1, and MEAF6-PHF1 fusions. Other fusion genes, including MBTD1-CXorf67, BRD8-PHF1, EPC2-PHF1, and EPC1-SUZ12, have been detected in LGESS (17). Nevertheless, it is worth noting that approximately one-third of LGESS cases, including this case, do not exhibit any identified fusion genes. This implies the possible presence of other undefined genetic molecular alterations (18).

Compared with LGESS, HGESS has a worse prognosis and more aggressive biological behavior. It has a recurrence rate of one year. Clinically, it has similarities with LGESS. Pathologically, HGESS has a uniform population of round and/or spindle-shaped cells, occasionally including low-grade spindle cells, displaying moderate-to-severe nuclear atypia (>10/10 high-power fields). In addition, focal components of LGESS may be observed. In immunohistochemistry, HGESS typically does not exhibit expression of CD10, ER, PR, and smooth muscle markers. Nevertheless, cyclin D1, a cell cycle protein, displays a diffuse marked positivity (>70% of cell nuclei). Furthermore, HGESS shows a high Ki-67 proliferation index. One study demonstrated that approximately half of HGESS cases may have a low-grade spindle cell component (19). In addition, there are some instances where HGESS can exhibit positive staining for ER, PR, and CD10 (18). HGESS cases without *YWHAE* gene rearrangement may show weak or even negative staining for cyclin D1 (20). Research suggests that positive nuclear staining of the β-catenin protein may serve as an immunohistochemical marker for HGESS (21). As for molecular diagnostics, fusion genes such as *YWHAE-NUTM2A*, *YWHAE-NUTM2B*, *ZC3H7B-BCOR*, and *BCOR ITD* have been observed. No fusion genes associated with HGESS were detected in this particular case.

Compared with LGESS, HGESS has a worse prognosis and more aggressive biological behavior. It has a recurrence rate of one year. Clinically, it has similarities with LGESS. Pathologically, HGESS has a uniform population of round and/or spindle-shaped cells, occasionally including low-grade spindle cells, displaying moderate-to-severe nuclear atypia (>10/10 high-power fields). In addition, focal components of LGESS may be observed. In immunohistochemistry, HGESS typically does not exhibit expression of CD10, ER, PR, and smooth muscle markers. Nevertheless, cyclin D1, a cell cycle protein, displays a diffuse marked positivity (>70% of cell nuclei). Furthermore, HGESS shows a high Ki-67 proliferation index. One study demonstrated that approximately half of HGESS cases may have a low-grade spindle cell component (19). In addition, there are some instances where HGESS can exhibit positive staining for ER, PR, and CD10 (18). HGESS cases without *YWHAE* gene rearrangement may show weak or even negative staining for cyclin D1 (20). Research suggests that positive nuclear staining of the β-catenin protein may serve as an immunohistochemical marker for HGESS (21). As for molecular diagnostics, fusion genes such as *YWHAE-NUTM2A*, *YWHAE-NUTM2B*, *ZC3H7B-BCOR*, and *BCOR ITD* have been observed. No fusion genes associated with HGESS were detected in this particular case.

This patient sought medical attention for uterine fibroids and was unexpectedly diagnosed with ESS (**Supplementary Figure 2**: Development of the disease). The tumor displays highly invasive behavior, infiltrating the deep muscle layer near the serosal surface and involving the cervical canal. Its biological behavior is consistent with that of HGESS in terms of invasiveness. Cytologically, the tumor exhibits a coexistence of low-grade spindle-shaped cells and high-grade round cells. Furthermore, the presence of pathological nuclear division (8/10 high-power fields) is more consistent with the characteristics of HGESS. Immunohistochemically, the tumor is positive for CD10 and ER and weakly positive for PR (15). These findings are consistent with immunological expression of LGESS. However, it should be noted that in certain cases, HGESS can also exhibit positive staining for ER, PR, and CD10 (18). Considering the concurrent immunohistochemical expression of HGESS, such as low positivity for cyclin D1 and a Ki-67 proliferation index of approximately 40%, the diagnosis of HGESS with concurrent LGESS is plausible. Further precision of the diagnosis can be achieved by genetic profiling for validation.

Although fusion genes associated with HGESS were not detected in this case, a frameshift mutation in exon 20 of the *MED12* gene was identified in the patient. This mutation differs from most of the previously reported *MED12* mutations, which predominantly occur in exon 2 and intron 1 (22, 23). MED12 is a constituent of the mediator complex, which acts as a key regulatory factor for the transcription of numerous genes (24, 25). Somatic mutations in *MED12* have been identified in approximately 70% of uterine leiomyomas and 10% of leiomyosarcomas (26). Studies have shown that *MED12* mutations in fibroadenomas are associated with dysregulation of estrogen signaling (27). This suggests that *MED12* mutations may play a crucial oncogenic role in estrogen-related ESS (28). The study reveals that mutations in exon 2 of *MED12* have been identified in ESS carrying the *JAZF1-SUZ12* or *JAZF1-PHF1* fusion genes. These mutations may confer additional oncogenic advantages (28).

The *MED12* mutations identified in ESS are novel and lack distinct pathological characteristics. There is no evidence of an adverse prognosis associated with the mutation status. In this case, the presence of partially high-grade areas within a low-grade ESS in combination with the *MED12* gene mutation is considered. Thus far, there has been no recurrence observed during follow-up. Given the existence of other rare types of ESS with genetic features that are not yet widely recognized, continuous monitoring of the patient is necessary to further gain experience and knowledge.

## 4 Conclusions

This study presents a case report of incidentally discovered ESS, which was initially misdiagnosed as uterine leiomyoma with tumor metastasis to the inferior vena cava. The pathological diagnosis was a combination of both LGESS and HGESS. Surprisingly, molecular analysis did not reveal any mutations commonly associated with LGESS or HGESS but instead identified a rare *MED12* mutation within the ESS. The patient underwent total hysterectomy, bilateral salpingectomy, and resection of the metastatic lesions after consulting the gynecological and surgical specialists. Postoperative management included radiotherapy, chemotherapy, and hormone therapy. Currently, the patient shows no signs of recurrence, and her condition is being closely monitored during follow-up.

## Data availability statement

The original contributions presented in the study are included in the article/**Supplementary Material**. Further inquiries can be directed to the corresponding authors.

## Ethics statement

Written informed consent was obtained from the individual(s) for the publication of any potentially identifiable images or data included in this article.

## Author contributions

WH: Visualization, Writing – original draft, Writing – review & editing. TZ: Conceptualization, Methodology, Writing – original draft, Writing – review & editing. HW: Conceptualization, Investigation, Methodology, Project administration, Resources, Writing – review & editing. ZL: Formal analysis, Methodology, Visualization, Writing – review & editing. PZ: Formal analysis, Project administration, Resources, Supervision, Writing – review & editing. XW: Project administration, Resources, Writing – review & editing. SW: Conceptualization, Funding acquisition, Methodology, Project administration, Supervision, Writing – review & editing.

## Supplementary material

Supplementary Figure 1 Gene Mutations: **(A)** *MED12* gene frameshift mutation. **(B)** *RB1* gene copy number loss. **(C)** *VEGFA* gene missense mutation.

Supplementary Figure 2 Development of the disease: the progression of the condition.
