# Supplementary figures and images for: Intravenous metastasis of unexpected uterine sarcoma in the context of uterine fibroids: case report and literature review
Source: Front Oncol. 2024 Feb 15;14:1354032. doi: 10.3389/fonc.2024.1354032 (PMC10902127; doi:10.3389/fonc.2024.1354032)

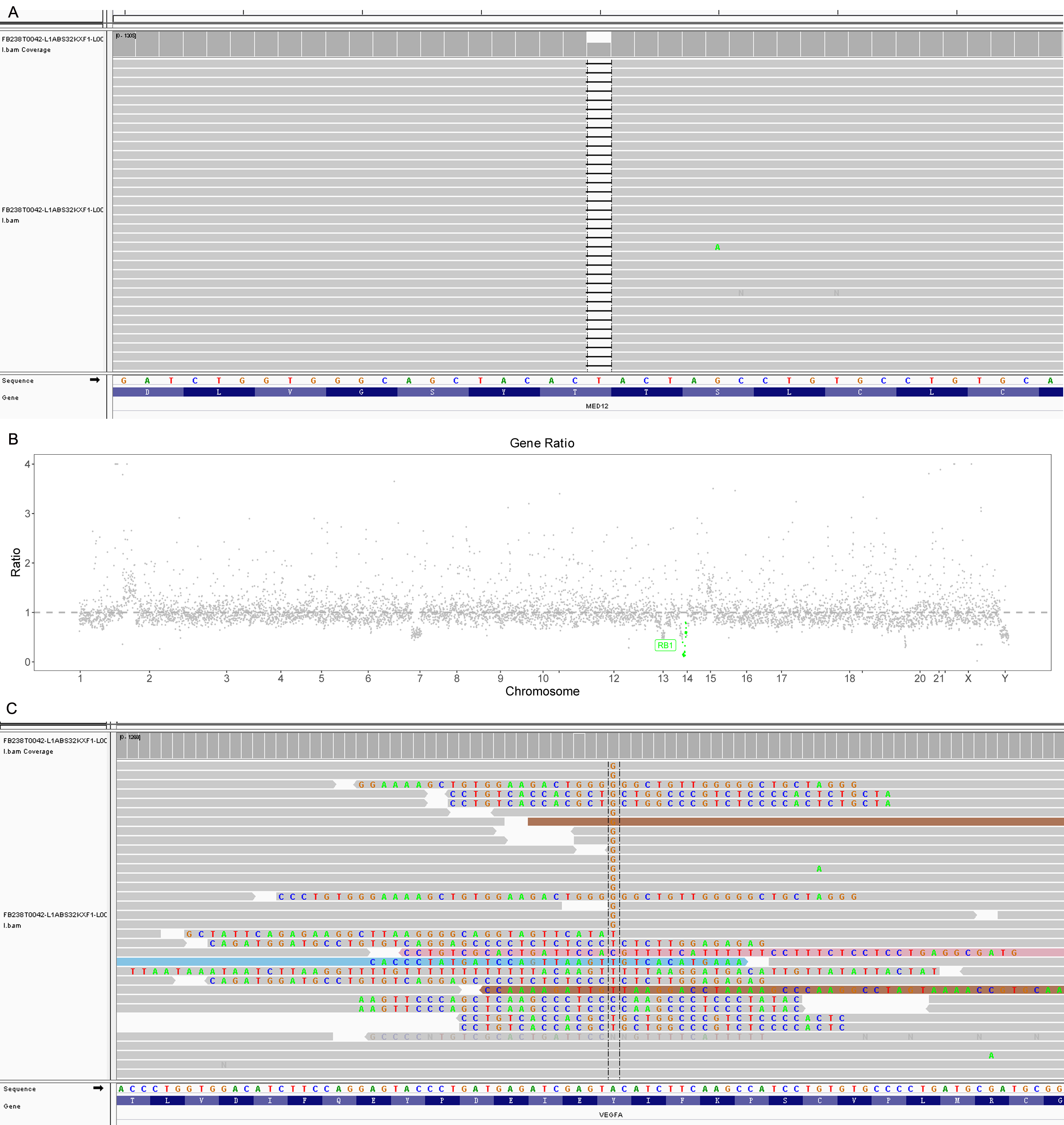

Supplement: Supplementary Figure 1 — Gene Mutations: (A) MED12 gene frameshift mutation. (B) RB1 gene copy number loss. (C) VEGFA gene missense mutation. [file Image_1.tif]

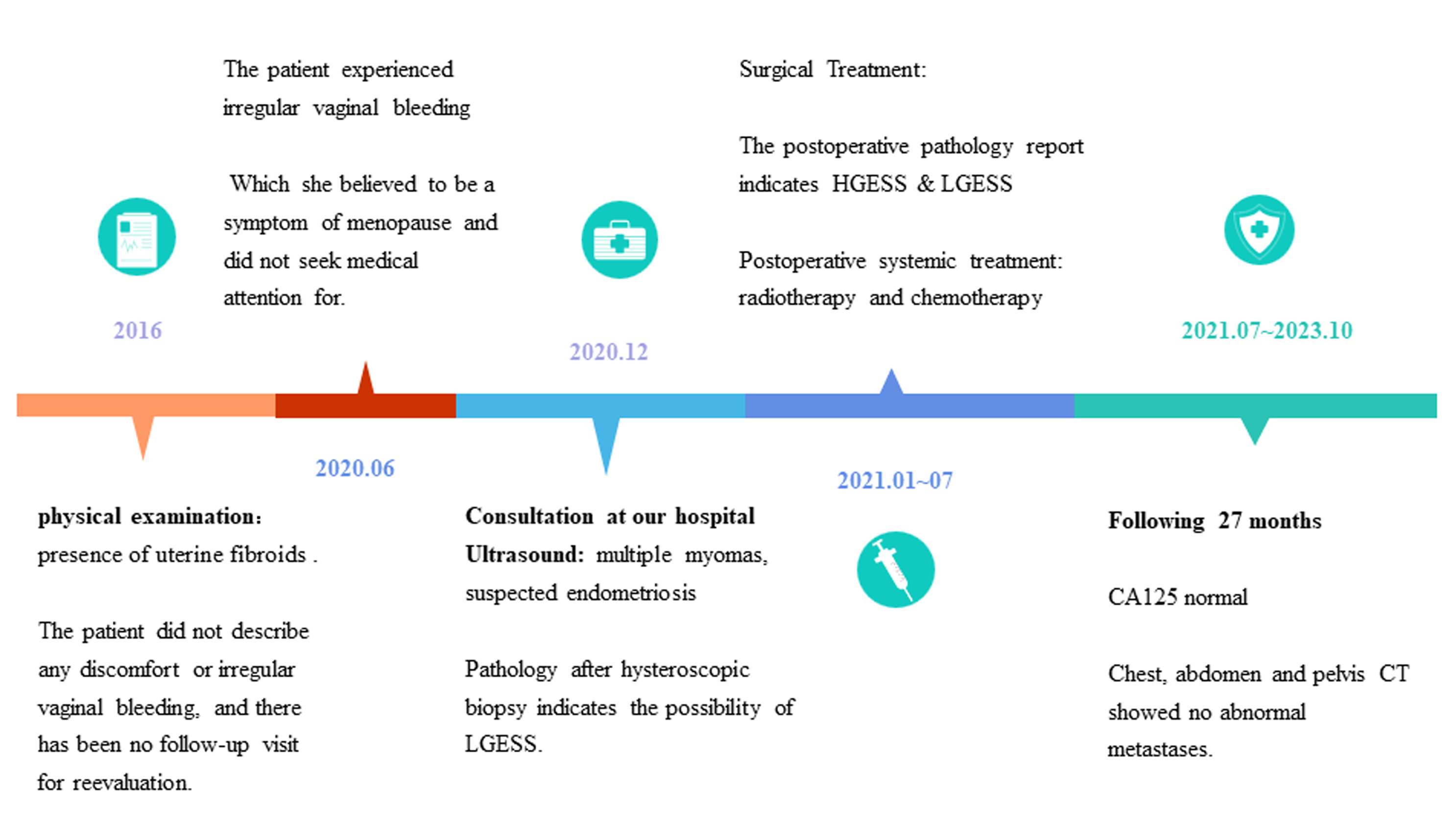

Supplement: Supplementary Figure 2 — Development of the disease: the progression of the condition. [file Image_2.tif]
